# America’s Cup Sailing: Effect of Standing Arm-Cranking (“Grinding”) Direction on Muscle Activity, Kinematics, and Torque Application

**DOI:** 10.3390/sports4030037

**Published:** 2016-06-27

**Authors:** Simon N. Pearson, Patria A. Hume, John Cronin, David Slyfield

**Affiliations:** 1 Sports Performance Research Institute New Zealand (SPRINZ), School of Sport and Recreation, Faculty of Health and Environmental Science, Auckland University of Technology, Private Bag 92006, Auckland 1020, New Zealand; patria.hume@aut.ac.nz (P.A.H.); john.cronin@aut.ac.nz (J.C.); david.slyfield@hpsnz.org.nz (D.S.); 2 Queensland Academy of Sport, QLD Sport & Athletics Centre, Kessels Rd, Nathan QLD 4111, Australia; 3 High Performance Sport New Zealand, PO Box 302 563, North Shore 0751, New Zealand

**Keywords:** yachting, biomechanics, movement analysis, performance

## Abstract

Grinding is a key physical element in America’s Cup sailing. This study aimed to describe kinematics and muscle activation patterns in relation to torque applied in forward and backward grinding. Ten male America’s Cup sailors (33.6 ± 5.7 years, 97.9 ± 13.4 kg, 186.6 ± 7.4 cm) completed forward and backward grinding on a customised grinding ergometer. In forward grinding peak torque (77 Nm) occurred at 95° (0° = crank vertically up) on the downward section of the rotation at the end of shoulder flexion and elbow extension. Backward grinding torque peaked at 35° (69 Nm) following the pull action (shoulder extension, elbow flexion) across the top of the rotation. During forward grinding, relatively high levels of torque (>50 Nm) were maintained through the majority (72%) of the cycle, compared to 47% for backward grinding, with sections of low torque corresponding with low numbers of active muscles. Variation in torque was negatively associated with forward grinding performance (*r* = −0.60; 90% CI −0.88 to −0.02), but positively associated with backward performance (*r* = 0.48; CI = −0.15 to 0.83). Magnitude and distribution of torque generation differed according to grinding direction and presents an argument for divergent training methods to improve forward and backward grinding performance.

## 1. Introduction

The America’s Cup is generally regarded as the most prestigious competition in sailing and is the oldest active trophy in international sport, dating back to 1851 [1]. On-water performance in America’s Cup competition is determined by numerous factors including tactics, crew work and yacht design, however, in terms of physical human performance during racing, grinding is considered to be the primary physical activity [2].

Grinding is a cyclic upper body task requiring the manual arm cranking of winches which control the movement of the mast and sails, making it a crucial component of manoeuvres such as tacking (turning on an upwind leg) and gybing (turning on a downwind) [3]. Grinding is performed in a standing position and the set-up of the winch system means that sailors are required to perform grinding in both a forward direction—pushing away from their body at the top of the rotation; and backward—pulling towards their body at the top of the rotation [2]. In race analysis of the 32nd America’s Cup and associated Louis Vuitton Challenger series, Neville, Calefato et al. [4] reported that an average of 20 tacks (5.5 ± 0.5 s in duration) and 8 gybes (11.2 ± 1.4 s) were performed during a race. These values were lower than previously reported figures of 30 tacks and 15 gybes per race [5]. However, it was calculated that when combined with mark roundings and the more frequent but less demanding grinding activity of sail trimming, a sailor whose principle role was grinding could expect a work to rest ratio of ~1:6 over an average 82 min race, and up to 1:3 in close racing. More accomplished crews (top four of the 11 challengers) completed manoeuvres in significantly shorter time than less accomplished crews [4], a finding that highlights the important relationship between grinding capability and overall race performance.

In addition to the performance aspects, grinders have been identified in a number of epidemiological studies as having the highest rate of injury amongst America’s Cup crew members [6,7,8,9,10]. Although these studies reported sometimes conflicting findings (possibly due to different methodologies), soft tissue injuries to the upper limb made up a large proportion of the preventable (non-accident/impact) injuries suffered by grinders, with the grinding activity itself directly attributed to 30% of injuries in this group [11]. A review of sailing injuries by Neville and Folland [10] suggested that poor technique and strength deficiencies may be risk factors when looking at the grinding task, and as such a better understanding of the muscles, movements, and loading patterns utilised may provide useful information for the preventative conditioning of sailors who perform grinding as part of their on-board role.

In recent years there has been an increasing body of research focussed on America’s Cup sailing from sport science and medicine perspective. With regards to the grinding activity in particular, studies examining the physiological [2,5] and strength and power [12] factors associated with performance, along with descriptions of the physical characteristics [4,13], nutritional requirements [14], and competition demands [4,15] have all been published. From a biomechanical perspective, an examination of grinding pedestal (crank-axle) height and crank length reported ground reaction force and kinematic data for forward grinding [16]. Results of this study indicated that while the standard crank length of America’s Cup yachts (250 mm) was near optimal for peak power generation, peak power was significantly greater for forward grinding at higher crank-axle heights (950–1150 mm) than was typically in use (850 mm). While this appears somewhat contradictory to results from seated arm-cranking which concluded that shoulder height relative to the crank axis has no influence on performance [17], Neville et al. identified substantial contribution of the legs to the standing arm cranking movement whereby restricting movement at the knees and ankles (achieved by joint splinting) reduced peak unilateral vertical ground reaction forces (indicating reduced lateral body mass transfer) and significantly increased the physiological strain [18]. Additional factors associated with improved power output through increased pedestal height were a more extended hip position (reduced forward trunk lean), and increased resultant ground reaction force, while it was noted that further examination of upper body function is required [16].

Away from the specific grinding or standing arm-cranking movement there have been a number of other papers on the kinematically similar seated arm cranking exercise, which is often used in rehabilitation and for wheelchair users [19]. Arm cranking papers have reported that hand-grip/forearm orientation affects muscle activation patterns [20], and that backward arm cranking is less proficient than forward, based on reduced kinematic variability at the elbow [21]. Possibly of most relevance, Bressel [19], in a comparison of forward and reverse (backward) seated arm cranking, observed little difference in either upper limb kinematics or oxygen consumption, but found that backward arm cranking required significantly greater activity of the biceps brachii, deltoid, and infraspinatus muscles.

While there may be some cross-over from the findings of this body of research, the differences in body position and posture due to the standing position adopted during America’s Cup grinding mean that there is justification in examining the mechanics of this activity in its own right. Concomitantly, of the current research examining the standing arm-cranking movement, so far none have examined both the forward and backward movements. The purpose of this study was therefore to describe the kinetic, kinematic, and muscular activation characteristics of the forward and backward grinding movements in America’s Cup sailing. It was hypothesised that the biomechanical requirements of the two movements would be substantially different, therefore necessitating different approaches to training.

## 2. Materials and Methods

### 2.1. Participants

Ten male America’s Cup sailors (33.6 ± 5.7 years, 97.9 ± 13.4 kg, 186.6 ± 7.4 cm) participated in this study by completing the grinding protocol. While the sailors varied in their primary role within the team, all performed grinding regularly as part of their on-board role. As maximal strength has previously been shown to be a key predictor of grinding performance, one-repetition maximum (1 RM) scores for the concentric only bench press (121.7 ± 26.1 kg) and bench pull (99.6 ± 17.1 kg) resistance strength training exercises was measured as an additional classification variable.

All procedures used in this study complied with the guidelines of the Auckland University of Technology Ethics Committee and had been granted ethical approval (reference 04/221).

### 2.2. Procedures: Performance Testing

**Figure 1:**
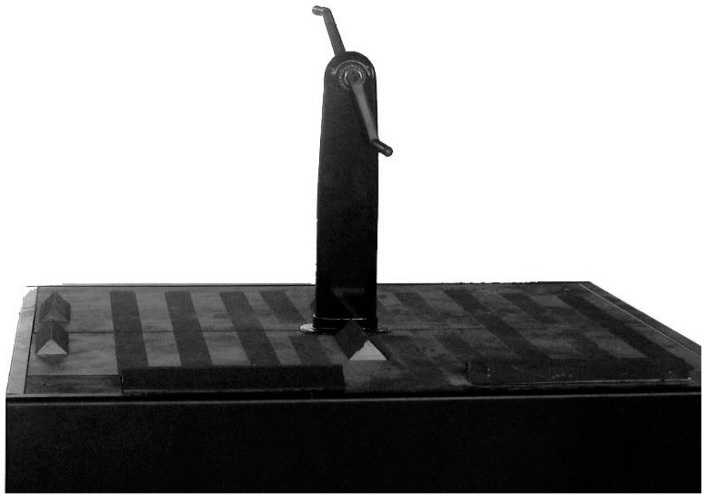
Ergometer for grinding performance testing.

Grinding performance testing was conducted on a custom-built grinding ergometer (Dynapack, Wellington, New Zealand) for which the technical specifics and reliability have previously been reported [22]. The ergometer was set up with standard pedestal (870 mm vertical) and crank arm (250 mm) dimensions for a main sheet grinding pedestal on an America’s Cup class yacht (see Figure 1).

Prior to testing, the sailors completed a self-determined warm-up on the grinding ergometer, typically consisting of 4–5 short bursts (5–10 s) of grinding, moving from low to high intensity, along with individually selected stretches. Performance testing consisted of two eight-second maximal-effort grinds for four conditions: Forward and backward directions, each performed against a moderate (48 Nm) and heavy (68 Nm) resistance. All trials were separated by a rest period of at least two minutes (representing a minimum 1:15 work:rest ratio). Testing loads were selected to correspond with moderate and heavy load conditions during on-water grinding manoeuvres, based on grinding speed ranges for a primary grinder. Testing conditions were presented in the following order: moderate-forward, moderate-backward, heavy-forward, heavy-backward; moving from what was generally perceived to be the easiest to hardest conditions in order to minimize the potential influence of any residual fatigue not removed by the two minute rest period. This was consistent with the protocol used regularly as part of the sailors general fitness monitoring and assessment, therefore meaning no specific familiarization was required for this testing.

Torque and velocity from both arms were recorded instantaneously by the ergometer. For the grinding performance measure raw power values were calculated by the Dynapack ergometer software using the formula: Power (W) = Torque × 2π × (rpm)/60. Raw power curves were smoothed using a second order recursive Butterworth low pass filter with a cut-off frequency of 6 Hz. Peak power and external work were calculated using a customised Labview (National Instruments, Austin, TX, USA) analysis program. Total work (J) performed during the five-second period following the attainment of peak power was determined for each trial, and the two-trial mean calculated. External work was used as the performance measure as this variable has been shown to be more reliable than peak power [22].

### 2.3. Procedures: Motion Analysis

Grinding for the motion analysis section of this study was conducted independently (within four weeks) of the grinding performance testing, although the protocol used was very similar. The primary point of difference was that the ergometer resistance was not set at an absolute level; rather, tests were performed against relative set loads customised to the individual sailor so that all participants were grinding within a similar speed range when grinding maximally (120–90 rpm), which incorporated the typically observed drop-off of 20–30 rpm during an eight-second trial. Individual loads were selected based on data from the medium and heavy load conditions in the performance testing. Those who typically met the speed range criteria at either of the standard testing loads (68 Nm, Heavy; 48 Nm, Moderate) were tested at those resistances, while those who didn’t meet those criteria were given an intermediate resistance of either 55 or 62 Nm, as judged most appropriate based on typical performance at the two standard loads. No participants were tested against a resistance higher or lower than this Moderate-Heavy range.

The decision to use relative loads was made due to the reasonably wide range of grinding abilities represented in the sample of interest to this study. As this section of study was focused on the mechanics of the movement rather than direct performance comparisons the desire was to examine grinding performance at high load (which has the biggest influence on boat performance) without inducing the degradation in basic technique that can be observed when an individual is performing at an excessively high load relative to their capability. If a set load was used for all participants, individuals either performing at loads much too easy or much too hard for their ability would likely have confounded the description of the grinding movement. As such, all individuals performed two maximal effort grinds in each of the forward and backward direction (four in total), against a moderate-heavy resistance, scaled relative to capability.

**Figure 2:**
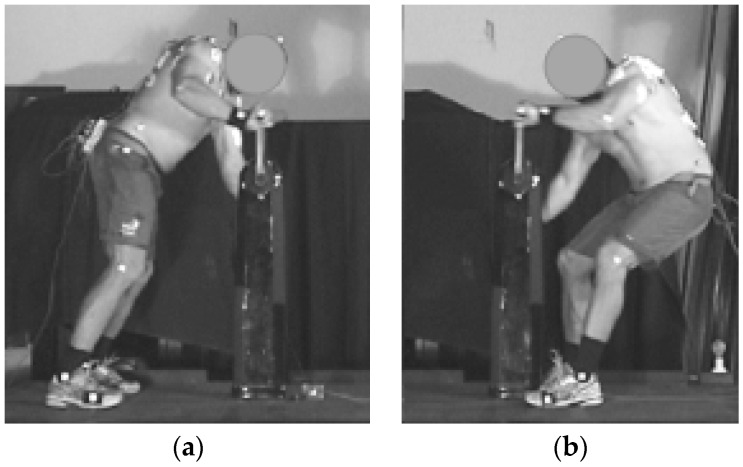
Examples of forward (**a**) and backward (**b**) body position at 0° crank angle.

**Figure 3:**
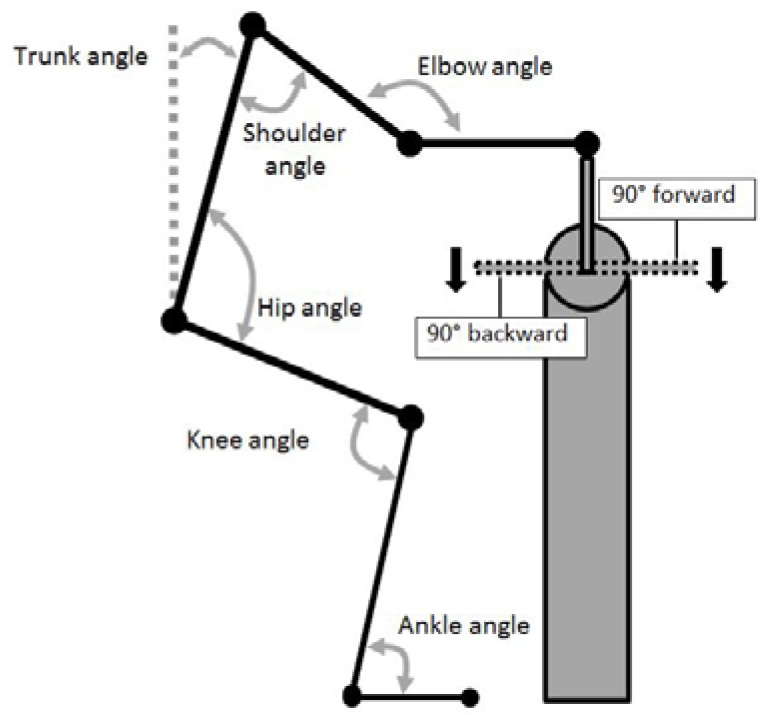
Analysis convention for joint angles and crank arm position, relative to sailor position.

Biomechanical data, consisting of muscle activation patterns using surface electromyography (EMG), torque applied to the grinding handles, and sagittal plane kinematics; were averaged over five complete grinding revolutions during the middle of the eight-second trial, avoiding the initial start/acceleration phase. All data were referenced based on handle motion relative to a 0° position where the handle vertically was above the hub (Figure 2) and angle moving positively in the direction of motion (Figure 3), which is consistent with conventions used in other arm cranking research [19].

EMG signals were collected using a Bortec AMT-8 system (Bortec, Calgary, Alberta, Canada) with pre-gelled Ag/AgCl surface electrodes, sampling at 1000 Hz. EMG activity data was collected on six upper body muscles: posterior deltoid, latissimus dorsi, triceps brachii (lateral head), anterior deltoid, pectoralis major (sternocostal head), and biceps brachii. Standard skin preparation was completed for electrodes placed according to the guidelines of Basmajian and Blumenstein [23]. Wires connected to electrodes were secured with tape to avoid movement artefacts. EMG data were full-wave rectified and low-pass filtered (15 Hz cut-off frequency). The signal from each muscle was normalised to a 100% scale of its own activation throughout the five-revolution analysis period, with 0% as the lowest recorded signal and 100% as the highest recorded signal. Muscle activation data was presented by dividing the grinding cycle into quartiles: upper (315°–44°), descending (45°–134°), lower (135°–224°), and ascending (225°–314°). Data are presented as the average relative activation during each quartile.

Torque application and angular position data were collected using an SRM Powermeter Science Road system (Schoberer Rad Messtechnik, Jülich, Germany) with torque analysis module sampling at 200 Hz. Characteristics of the captured torque-angle data output from the SRM system were such that averaging of the five cycles of interest was conducted without any additional smoothing. The SRM Powermeter was attached to the hub of the grinding pedestal using a custom-made mounting plate which enabled collection of data independently from a single crank arm, eliminating the issue of standard SRM mounting in bicycles, which captures combined torque from both cranks. Data was therefore captured from one crank arm and one side of the body only for any particular trial. As the grinding ergometer was optimized for hub rotation in a single direction, the single side analysis corresponded to the sailor’s right hand side for forward grinding and left hand side for backward grinding (see Figure 2).

Full body sagittal plane motion was recorded using a video camcorder (Sony DCR-TRV120E, Sony Corporation, Tokyo, Japan) recording at 50 fields/s with a 1/500 shutter speed. To aid digitisation, markers were placed on the distal head of the fifth metatarsal bone, lateral malleolus of the ankle, lateral condyle of the tibia, greater trochanter, lateral aspect of the acromion process, lateral epicondyle of the humerus, and the styloid process of the ulna. Two-dimensional sagittal plane relative joint angles in degrees (°) were obtained through digitisation using APAS (Ariel Dynamics, Trabuco Canyon, CA, USA) for the ankle, knee, hip, shoulder, and elbow (Figure 3). Shoulder angle was measured using the trunk as a relative 0° position, with a positive value moving anteriorly into flexion. Trunk angle was measured relative to vertical, with a positive value indicating the shoulder was anterior to the hip. Kinematic data were summarised using four reference points during the grinding cycle based on handle position: 0° = handle vertically above the point of rotation (hub); 90° = horizontal to the hub, moving from 0° in the direction of rotation (on the sailor’s side of the pedestal for forward grinding, away from the sailor for backward); 180° = vertically below the hub; 270° = horizontal to the hub, on the way back up towards the 0° position.

Data were synchronised using the onset of a five-volt pulse to the computer-based data acquisition system, generated simultaneously with the activation of an LED light in the line of view of the video camera. Due to the collection of torque-angle (SRM) data being limited to a single crank, all data were analysed for only the right side during forward grinding and the left side for backward grinding.

### 2.4. Statistical Analyses

Descriptive statistics for all variables are represented as mean and standard deviation (SD). The presence of significant systematic discrepancies between forward and backward grinding for given variables during the cycle were determined using a two-tailed paired *t*-test with alpha set at *p* < 0.05. In addition to descriptive data the relationship between variability in torque application throughout a revolution and grinding performance was also examined using Pearson correlation analysis. Grinding performance (J) for an individual was log transformed (ln) and corrected for maximal strength (1RM—also log transformed), with the residuals from this correction plotted against the sailor’s variation in torque application, as represented by the standard deviation of the log-transformed SRM data. To make inferences about true (population) values of the relationship, the uncertainty in the effect was expressed as likelihoods that the true value of the effect represents substantial change (harm or benefit) using the methods of Hopkins [24], with the smallest worthwhile change set as 0.10 standardised Cohen units [25] and confidence limits set at 90%.

## 3. Results

### 3.1. Torque Application and Grinding Performance

**Figure 4:**
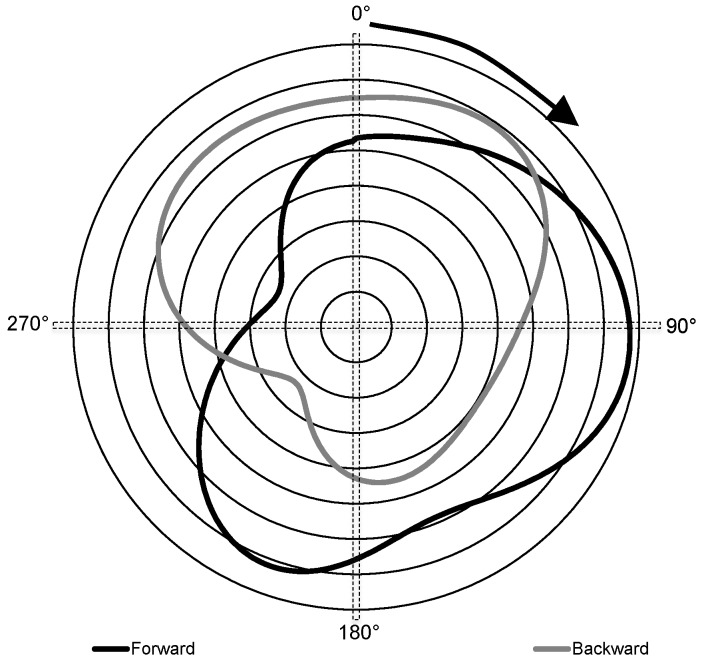
Average torque-angle curves for forward (black) and backward (grey) grinding. Torque increases as the traces move away from the centre (lines = 10 Nm increments). Arrow indicates the direction of handle rotation. 0° = crank handle vertically upwards from the centre.

Forward grinding produced significantly more work and higher mean torque throughout the cycle than backward grinding (Table 1). Greatest torque application occurred through 60°–200° for forward grinding and 300°–40° for backward grinding (see Figure 4). Peak torque for the group average torque-angle data was 77.5 ± 4.1 Nm at 95° for forward grinding and 68.8 ± 2.9 Nm at 35° for backward grinding. Variation in torque application throughout the grinding cycle was negatively associated with strength corrected forward grinding performance (*r* = −0.60; *p* = 0.09; 90% CI = −0.88 to −0.02) but positively associated with backward performance (*r* = 0.48; *p* = 0.19; 90% CI = −0.15 to −0.83). While these relationships were non-significant, inference-based statistics, which take into account the population size and confidence limits, indicate that it was likely or probable that these correlations represented clinically meaningful relationships.

### 3.2. Joint Kinematics

When grinding forward the sailors typically stood more upright and leaned over the grinding pedestal when compared to backward grinding (see Figure 2), as indicated by significantly less hip flexion and more forward trunk lean throughout the cycle (see Table 2). Sailors also tended to have a more plantar flexed ankle and greater knee flexion during backward grinding. Elbow and shoulder angles differed significantly at 90° and 270° but tended to be more similar at 0° and 180°.

### 3.3. Muscle Activation

**Figure 5:**
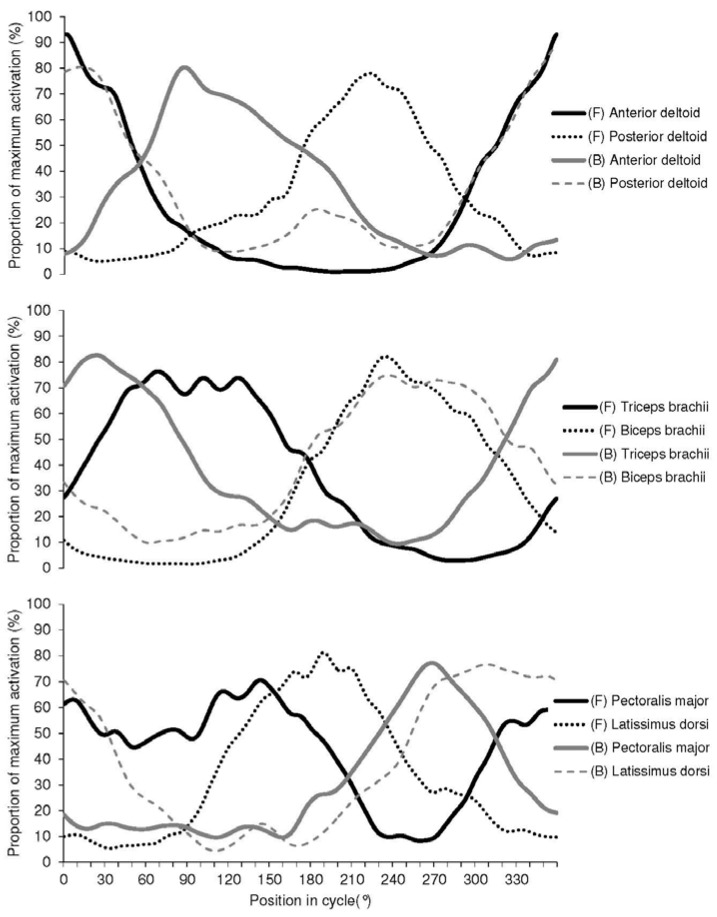
Average EMG activity patterns during forward (F) and backward (B) grinding for 10 grinders. Crank angle defined as 0° when positioned vertical and above the pedestal. Crank angles are positive in the direction of motion.

EMG activity patterns for forward and backward grinding are presented numerically in Table 3 and graphically in Figure 5, with muscles paired according to approximate agonist-antagonist relationships based on typical involvement in joint actions. In backward grinding there was a high level of activation (>50%) from five of the six muscles monitored during the ascending (pectoralis major, latissimus dorsi, and biceps brachii) and upper (latissimus dorsi, posterior deltoid, and triceps brachii) quartiles of the rotation where torque increased rapidly and peaked before declining again as the crank arm came in close to the sailors body at ~90°. Through the descending and lower quartiles of the cycle, as the crank arm passed down to its lowest point (180°) and then started moving away from the sailor the only notable activity was from smaller muscles—anterior deltoid and the triceps and biceps brachii. At the point of minimum backward grinding torque (227°, 23 Nm), biceps brachii was the only muscle showing any meaningful level of activation, with pectoralis major and latissimus dorsi just beginning to fire.

In contrast, forward grinding showed a much more even spread of muscle activity throughout the rotation, with anterior deltoid, pectoralis major and triceps brachii dominant through the upper and descending quartiles (315°–134°) and latissimus dorsi, biceps brachii, pectoralis major and posterior deltoid highly active through the majority of the lower and ascending phases. As with backward grinding, the point of minimum torque application for forward grinding (294°, 24 Nm) was on the ascending section and corresponded with a period during which only biceps brachii was notably active.

## 4. Discussion

This study provided torque-angle curves for upper limb arm grinding for America’s Cup sailors, along with kinematic and muscle activation data characterizing the movement. Cycling literature [26,27,28] has consistently reported that torque or applied force for a single limb occurs almost entirely in the down stroke (0°–180°, with 0° crank position again vertically upward) with virtually no positive force, and in many cases a negative force applied during the following up stroke [27,28,29]. In contrast torque during grinding was never negative, with the mean curve remaining above 20 Nm throughout the entire 360° cycle. The torque application differed between forward and backward grinding. While backward grinding followed a similar pattern to cycling in which the majority of torque was applied through half of the rotation (270°–80°) with a considerable drop-off through the remaining half-circle, in forward grinding a high level of force at the handles was maintained through a much greater proportion of the rotation (340°–240°), leaving only a relatively small “dead spot” of ~100° in which torque was reduced. This was in contrast to cycling literature, with Neptune, Kautz and Zajac [29] observing very little difference in tangential pedal force between forward and backward pedalling. A potential reason for the difference in torque throughout the cycle may relate to foot position adopted, which in the case of forward grinding is away from the pedestal, characterised by a relatively dorsiflexed ankle and forward trunk lean, enabling notable contribution from the lower limb and body mass [16,18]. In contrast, the position adopted in backward grinding is much less balanced with the feet close to the grinding pedestal, greater flexion at the hips and more upright trunk, which results in effectively “hanging” off the handles, which is likely to limit the ability to apply force to the handles consistently.

Relative levels of torque throughout the rotation were also reflected in muscle activation patterns. On an individual basis muscle activation patterns were consistent with the bi-phasic nature of grinding with distinct periods of activation and in-activation, which has also been seen previously in seated arm cranking [19]. However, the spread of muscles active at any one time differed with grinding direction, with more consistent contributions from the muscles examined (most notably latissimus dorsi and pectoralis major) throughout the forward grinding cycle compared to backward.

In addition to the importance of identifying causes of low torque zones or “dead spots” which can impact on performance, it is also of interest to examine where grinding biomechanics are optimum. In terms of maximum force application, both peak and mean torque were ~12% higher for forward grinding than for backward grinding, which was consistent with previous findings where work performed over five seconds of maximal grinding was 12%–15% greater in the forward direction [22]. Fujii and Nagasaki [21] concluded that reverse arm cranking was less proficient than forward arm cranking. In the average torque-angle curve for the cohort, peak torque occurred at 95° and 35° crank angles for forward and backward grinding respectively, aligning with the end of shoulder horizontal flexion and elbow extension (push movement) in forward grinding, and at the completion of shoulder extension and elbow flexion (pull movement) in backward grinding. Although forward grinding has a more even distribution of torque throughout the cycle, the location of the peaks does support the view that the primary “work generating” movements are upper body push and pull for forward and backward grinding respectively.

The importance of the areas of maximum torque application to the respective grinding directions also appears to differ. Analysis of the relationship between torque application and strength-corrected grinding performance (removing strength as a covariate to focus on technical performance) showed that greater variation in torque throughout the grinding cycle was positively associated with backward performance (*r* = 0.48). Although not conclusive, this does indicate some justification in focusing on the primary pull movement (maximising the peak) to improve backward grinding performance. In contrast, the negative association between torque variability and forward grinding performance (*r* = −0.60) suggest that improvements in forward grinding are likely to be achieved through the ability to maintain torque throughout the entire cycle. These findings also align with previous research where maximum muscular force was related to forward grinding performance, while power and rate of force development were important for backward grinding [12,30]. While maximal strength and force were important in both directions, the additional importance of the speed of force generation for backward grinding was consistent with the torque profile observed in this study with cyclic loading, unloading, and re-loading having to be repeated during a short period of time.

Limitations of the study include whether the two minutes for rest period was long enough for recovery given metabolic disturbance from the maximal 8-s effort, and whether there is the effect of limb dominance may have influences the findings. However, given the magnitude of the differences observed, it seems unlikely that the observed patterns would be fundamentally different, although the strength of the relationships may well be altered. It should also be noted from a kinematic perspective, that although the majority of the grinding movement occurs in the sagittal plane, three dimensional analysis would improve accuracy of some of the measures taken in the upper body (elbow, shoulder and trunk). The use of absolute loads rather than relative loads could also be considered given competition involves high load maximal effort grinding and inevitably some breakdown of technique. Further investigations could consider the use of force plates to better understand lower limb contribution to backward grinding, while from an injury perspective a prospective investigation could provide valuable insight into whether movement or torque application patterns predispose sailors to certain types of injuries.

## 5. Conclusions

Overall, the disparities observed between the biomechanical characteristics of forward and backward grinding are somewhat at odds with previous literature from cycling [29] and seated arm-cranking [19] which observed relatively little directional difference. While the discrepancy with cycling can be explained by the substantial differences in lower limb structure and function, the same does not apply for seated arm cranking. It would seem that the additional degrees of positional freedom along with the potentially greater contribution of body weight allowed by the standing position used in grinding makes a difference to the variation in biomechanics between cranking direction. This is observable at the most basic level by the different postural positions selected by the sailors when grinding backward *versus* forward, as compared to the relatively fixed position in seated arm cranking.

In terms of specific information that could potentially be used to improve performance, we have identified the movements and muscles involved in the grinding activity and the factors involved in periods of high and low torque. In many sporting movements it is the reduction in inefficiencies as much as maximum capability that determines overall proficiency and so it may be that working to improve the areas of weakness in the grinding cycle, whether through conditioning or technical changes, can improve grinding performance. However, further investigation would be required to determine whether this is in fact a practical solution or if it is the result of an unchangeable factor such as structurally limited function of the upper limb in that position. Even within the findings of this study, there is evidence that the influence of biomechanical factors on performance differ according to direction, and as such any intervention should be customised for forward and backward grinding.

## Figures and Tables

**Table 1:** Summary data (Mean ± SD) for grinding performance, mean torque application and variability in torque application.

Direction	Grinding Performance (J)	Torque Application	
		Mean (Nm)	Variability (SD of ln (J))
Fwd	2938 ± 317	56.0 ± 10.9	38.3 ± 5.7
Back	2738 ± 316	49.9 ± 10.2	35.6 ± 13.4
*Diff in Mean (%)*	7.3% *	12.2% *	*7.5%*

* Significant difference between forward and backward grinding (*p* < 0.05).

**Table 2:** Average (±SD) joint angles for forward (Fwd) and backward (Back) grinding presented at four positions (in degrees) during the cycle. Position (°) increases in the direction of motion.

*Position*	Direction	Ankle	Knee	Hip	Shoulder	Elbow	Trunk
*0°*	Fwd	103 ± 11 *	136 ± 12	133 ± 5 *	15 ± 15 *	93 ± 19	39 ± 4 *
	Back	136 ± 8 *	134 ± 9	101 ± 6 *	39 ± 5 *	100 ± 14	26 ± 5 *
*90°*	Fwd	98 ± 9 *	134 ± 15	128 ± 8 *	72 ± 10 *	166 ± 7 *	46 ± 6 *
	Back	130 ± 10 *	131 ± 4	106 ± 5 *	−1 ± 9 *	92 ± 7 *	20 ± 5 *
*180°*	Fwd	102 ± 9 *	139 ± 12 *	122 ± 6 *	51 ± 9	154 ± 8	50 ± 4 *
	Back	124 ± 9 *	122 ± 4 *	94 ± 11 *	47 ± 9	162 ± 7	33 ± 7 *
*270°*	Fwd	114 ± 13	150 ± 14 *	133 ± 7 *	2 ± 7 *	105 ± 9 *	44 ± 3 *
	Back	127 ± 8	123 ± 5 *	97 ± 8 *	73 ± 10 *	162 ± 15 *	30 ± 6 *

* Significant difference between forward and backward grinding (*p* < 0.05).

**Table 3:** Mean (±SD) relative muscle activation (% of maximum) per quartile of the grinding cycle for forward (Fwd) and backward (Back) grinding. Dark grey shading indicates mean activation over 60%, light grey shading indicates 40%–60%.

*Quartile*		PD	LD	TB	AD	PM	BB
*Upper*	Fwd	19 ± 7	15 ± 2	20 ± 9	72 ± 10	44 ± 12	35 ± 16 *
*(315°–44°)*	Back	72 ± 11	64 ± 11	71 ± 11	16 ± 10	22 ± 10	34 ± 12 *
*Descending*	Fwd	12 ± 2	21 ± 12	63 ± 14	38 ± 17	56 ± 4	7 ± 2
*(45°–134°)*	Back	24 ± 15	14 ± 9	48 ± 17	65 ± 13	13 ± 2	13 ± 2
*Lower*	Fwd	31 ± 15	67 ± 10	43 ± 15	6 ± 3	58 ± 5	33 ± 18
*(135°–224°)*	Back	18 ± 5	15 ± 7	18 ± 3	44 ± 13	23 ± 11	42 ± 18
*Ascending*	Fwd	52 ± 5	45 ± 17	14 ± 5	19 ± 13	20 ± 9	76 ± 5
*(225°–314°)*	Back	21 ± 13	59 ± 17	19 ± 10	11 ± 3	64 ± 9	71 ± 4

Muscles presented are: Posterior deltoid (PD), latissimus dorsi (LD), triceps brachii (TB), anterior deltoid (AD), pectoralis major PM), and biceps brachii (BB); Comparisons between grinding direction were significant for all muscles in all quartiles other than biceps brachii in the upper quartile (shaded grey).

## References

[1] Rayner R., Thompson T. (1996). The Story of the America’s Cup 1851–1995.

[2] Neville V., Pain M.T., Folland J.P. (2009). Aerobic power and peak power of elite America’s Cup sailors. Eur. J. Appl. Physiol..

[3] Whiting P. (2007). The 32nd America’s Cup: A Simple Guide.

[4] Neville V., Calefato J., Perez-Encinas C., Rodilla-Sala E., Rada-Ruiz S., Dorochenko P., Folland J.P. (2009). America’s Cup yacht racing: Race analysis and physical characteristics of the athletes. J. Sports Sci..

[5] Bernardi M., Quattrini F.M., Rodio A., Fontana G., Madaffari A., Brugnoli M., Marchetti M. (2007). Physiological characteristics of America’s Cup sailors. J. Sports Sci..

[6] Allen J.B. (2005). Sports medicine injuries in the America’s Cup 2000. N. Z. J. Sports Med..

[7] Hadala M., Barrios C. (2009). Different strategies for sports injury prevention in an America’s Cup yachting crew. Med. Sci. Sports Exerc..

[8] Hadala M., Barrios C. (2009). Sports injuries in an America’s Cup yachting crew: A 4-year epidemiological study covering the 2007 challenge. J. Sports Sci..

[9] Neville V., Molloy J., Brooks J.H., Speedy D.B., Atkinson G. (2006). Epidemiology of injuries and illnesses in America’s Cup yacht racing. Br. J. Sports Med..

[10] Neville V., Folland J.P. (2009). The epidemiology and aetiology of injuries in sailing. Sports Med..

[11] Allen J.B., De Jong M.R. (2006). Sailing and sports medicine: A literature review. Br. J. Sports Med..

[12] Pearson S.N., Cronin J.B., Hume P.A., Slyfield D. (2009). Strength and power determinants of grinding performance in America’s Cup sailors. J. Strength Cond. Res..

[13] Pearson S.N., Hume P.A., Mellow P., Slyfield D. (2005). Anthropometric dimensions of Team New Zealand America’s Cup sailors. N. Z. J. Sports Med..

[14] Bernardi E., Delussu S.A., Quattrini F.M., Rodio A., Bernardi M. (2007). Energy balance and dietary habits of America’s Cup sailors. J. Sports Sci..

[15] Neville V., Gant N., Folland J.P. (2009). Thermoregulatory demands of Elite Professional America’s Cup Yacht Racing. Scand. J. Med. Sci. Sports.

[16] Neville V., Pain M.T., Kantor J., Folland J.P. (2010). Influence of crank length and crank-axle height on standing arm-crank (grinding) power. Med. Sci. Sports Exerc..

[17] Cummins T.D., Gladden L.B. (1983). Responses to submaximal and maximal arm cycling above, at, and below heart level. Med. Sci. Sports Exerc..

[18] Neville V., Zaher N., Pain M.T., Folland J.P. (2009). Lower limb influence on standing arm-cranking (“grinding”). Int. J. Sports Med..

[19] Bressel E., Heise G.D. (2004). Effect of arm cranking direction on EMG, kinematic, and oxygen consumption responses. J. Appl. Biomech..

[20] Bressel E., Bressel M., Marquez M., Heise G.D. (2001). The effect of handgrip position on upper extremity neuromuscular responses to arm cranking exercise. J. Electromyogr. Kinesiol..

[21] Fujii N., Nagasaki H. (1995). Efficiency and proficiency of bimanual cranking: Differences between two cranking patterns. Percept. Mot. Skills.

[22] Pearson S.N., Hume P.A., Slyfield D., Cronin J.B. (2007). External work and peak power are reliable measures of ergometer grinding performance when tested under load, deck heel and grinding direction conditions. Sports Biomech..

[23] Basmajian J.V., Blumenstein R. (1980). Electrode Placement in EMG Biofeedback.

[24] Hopkins W.G. Calculating likely (confidence) limits and likelihoods for true values (Excel spreadsheet). http://sportsci.org/resource/stats/xcl.xls.

[25] Cohen J. (1988). Statistical Power Analysis for the Behavioral Sciences.

[26] Bertucci W., Grappe F., Girard A., Betik A., Rouillon J.D. (2005). Effects on the crank torque profile when changing pedalling cadence in level ground and uphill road cycling. J. Biomech..

[27] Neptune R.R., Hull M.L. (1998). Evaluation of performance criteria for simulation of submaximal steady-state cycling using a forward dynamic model. J. Biomech. Eng..

[28] Reiser R.F., Peterson M.L., Broker J.P. (2003). Instrumented bicycle pedals for dynamic measurement of propulsive cycling loads. Sports Eng..

[29] Neptune R.R., Kautz S.A., Zajac F.E. (2000). Muscle contributions to specific biomechanical functions do not change in forward *versus* backward pedaling. J. Biomech..

[30] Pearson S.N., Cronin J.B., Hume P.A., Slyfield D. (2009). Effects of a power-focussed resistance training intervention on backward grinding performance in America’s Cup sailing. Sports Biomech..

